# Telemedicine monitoring in the follow-up of kidney transplant recipients: consensus indications from an Italian panel of surgeons and nephrologists after the COVID-19 experience

**DOI:** 10.1007/s40620-021-01193-w

**Published:** 2022-02-17

**Authors:** Luigi Biancone, Enrico Minetti, Paride De Rosa, Paolo Rigotti, Giovanni Stallone, Marco Volpe, Franco Citterio

**Affiliations:** 1 Department of Medical Sciences, University of Turin and A.O.U. Città Della Salute E Della Scienza Di Torino, Turin, Italy; 2 S.C. Nephrology, ASST Grande Ospedale Metropolitano Niguarda, Milan, Italy; 3 General Surgery and Kidney Transplantation Unit, ”San Giovanni Di Dio E Ruggi D’Aragona” University Hospital, Scuola Medica Salernitana, Salerno, Italy; 4 Renal and Pancreas Transplant Unit, Hospital‒University of Padua, Padua, Italy; 5 Nephrology, Dialysis and Transplantation Unit, Department of Medical and Surgical Science, University of Foggia, Foggia, Italy; 6 Business Integration Partners SpA, Milano, Italy; 7 Department of Surgery, Renal Transplantation Unit, Fondazione Policlinico Universitario A. Gemelli, IRCCS, Rome, Italy

**Keywords:** Kidney transplant recipient, Follow-up, Telemedicine, Video visit

## Abstract

The quality of follow-up has clearly emerged as a key factor for long-term kidney graft survival. Currently, many clinics are facing difficulties in delivering optimal surveillance because of the increased number and complexity of kidney transplant recipients, and because of the COVID-19 pandemic. Additional ways of performing follow-up visits are needed and telemedicine has emerged as a tool to strengthen patient care intensity. Six Italian transplant surgeons and nephrologists convened via teleconference to develop a consensual model of video visits for the follow-up of kidney transplant recipients. Issues discussed were: profile of eligible patients; assessments that can be carried out; video visit organization and medical professionals involved; supporting tools and implementation. The video visit was consensually recognized as the most relevant for the follow-up of kidney transplant recipients. Eligible patients should have basic electronic devices and the skills to correctly use them and be in clinically stable condition. With the exception of physical and instrumental examination, and kidney biopsy, all other assessments are feasible during a video visit and can be implemented by specific training and use of supporting tools. The video visit model is simple and adaptable to most transplant patients. It is not intended to replace face-to-face examinations, but is an additional tool for improving the intensity of follow-up of kidney transplant recipients, which can be integrated into current monitoring protocols.

**Graphical abstract:**

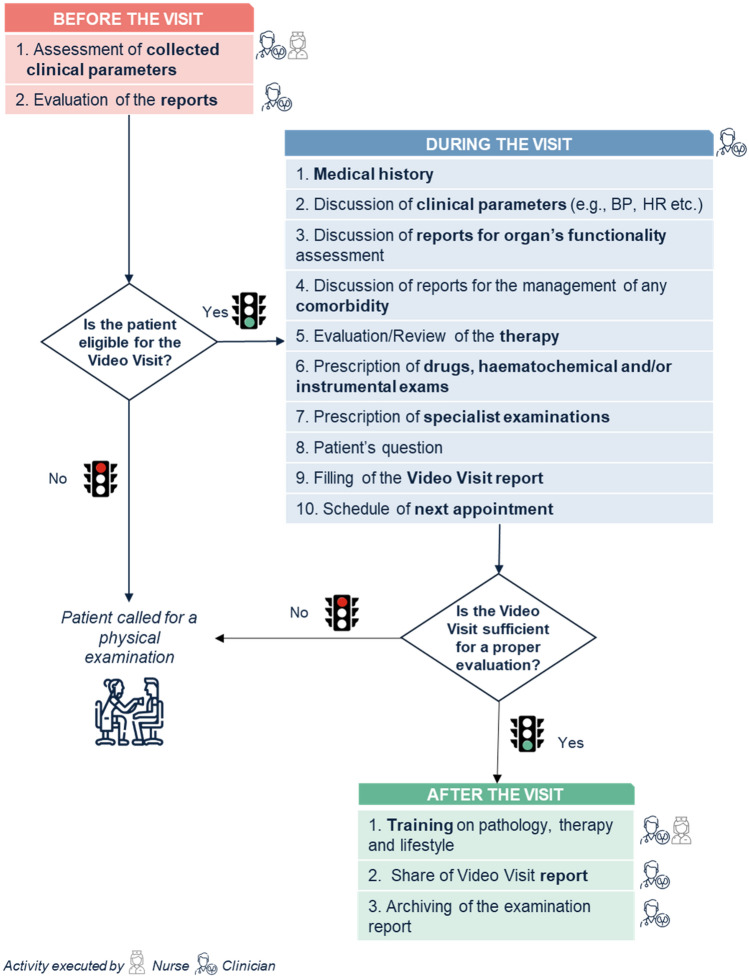

## Introduction

Kidney transplant recipients need life-long follow-up after transplantation to avoid acute graft rejection and opportunistic infections, optimize renal function, ensure compliance to prescribed treatments, and prevent the long-term complications of immunosuppressive therapy [1]. The recommended frequency of follow-up visits is several times weekly during the first months, every 2–6 weeks after 4–12 months, and every 3–6 months thereafter [1]. As the number and complexity of kidney transplant recipients is steadily growing, many transplant and nephrology clinics are facing increasing difficulties in delivering optimal and timely surveillance services [2]. The burden placed on transplant and nephrology clinics is considerable also because resources have generally remained unchanged. Alternative ways of following-up with kidney transplant recipients are therefore urgently needed.

The healthcare crisis generated by the outbreak of Coronavirus disease 2019 (COVID-19) has further complicated the follow-up of kidney transplant recipients, thus increasing the need for alternatives to conventional face-to-face medical visits. Routine follow-up has become more difficult due to the allocation of resources to the COVID-19 emergency. In addition, the discontinuation of in-person routine medical services for safety reasons and elevated risks for transplant recipients attending medical clinics has further complicated this process [3, 4]. Furthermore, it is common “real life” experience that, besides onsite visits, more and more patients need to have additional contacts with the physicians and nurses of the transplant team: these patient-promoted contacts occur through phone calls, faxes, emails and phone text/voice messages, tools that often may be inappropriate for the complexity of certain health issues and/or have drawbacks in terms of privacy regulations. Telemedicine—the remote delivery of medical care using information and communication technologies—has emerged as a viable alternative to in-person visits, and recommendations on how to manage patients remotely have been issued by associations and clinicians from many therapeutic areas [5–8].

Overall, the quality of follow-up has clearly emerged as a key factor for long-term graft survival [9, 10] by comparing kidney transplant outcomes among different health-care systems. In addition, the lack of compliance to immunosuppressive therapy is now recognized as a major determinant for graft failure [9, 10] due to chronic rejection, which is the leading cause of graft loss. Thus, improving the intensity of patient contact also in the long-term is highly recommended for monitoring compliance and preventing non-adherence issues.

Prompted by the increasing organizational burden on transplant and nephrology clinics and the strain caused by the COVID-19 pandemic on the national healthcare system, a board of six Italian transplant surgeons and nephrologists convened in a cycle of teleconferences to discuss the potential of telemedicine for follow-up appointments with kidney transplant recipients. The ultimate goal was to design a consensual model suggesting the “best practice” of virtual video visits, to be integrated into the current management of kidney transplant recipients. Considering current telemedicine regulations in Italy, relevant issues concerning the video visit, the profile of kidney transplant recipients, assessments that can safely be performed remotely, and the required infrastructures, here we report the consensus-based model of video visits.

## Methods

During 2020, six transplant surgeons and nephrologists from six different Italian regions convened in a scientific webinar to address post-operative monitoring of kidney transplant recipients in the current situation of work overload and general healthcare crisis generated by the COVID-19 pandemic. The panelists were selected given their expertise, being either transplant surgeons or nephrologists, directors of the main kidney Transplant Centers in Italy, with additional experience in patient management with telemedicine. Furthermore, the project team was supported by members of the consultancy firm BIP (https://www.bipconsulting.com/en-uk/) with expertise in data analysis and modeling for developing patient pathway systems including digitalization. After an initial discussion among the panel of experts to define the main points, the (agreed) results were shared with 25 Italian centers during three boards, to meet a general agreement. The main objective of the scientific board was to explore the potential of telemedicine for the remote management of transplanted patients, and to design the best practice for a telemedicine-based follow-up visit in the form of a video visit. To this end, the advisory board first analyzed existing telemedicine services in Italy. Then, based on published literature and the direct experience of the board members, four relevant issues related to the video visit were discussed: (1) profile of patients eligible for the video visit; (2) potential assessments performed during the video visit; (3) video visit organization, nurse and medical professionals’ training and involvement; (4) implementation of supporting tools.

## Features of telemedicine for the follow-up of kidney transplant recipients

Among the various medical services encompassed by telemedicine, the video visit was consensually recognized as the most relevant for the follow-up of kidney transplant recipients. The expected advantages, according to the board, are manifold and are summarized in Table 1. Remote monitoring may help clinicians to optimize the time and resources needed to improve the management of visits and the intensity of the follow-up, thus resulting in more efficient practices. Theoretically, continuous monitoring should be easier to ensure in an emergency situation as well. From the patient’s perspective, less traveling for routine visits could significantly simplify monitoring, especially in terms of time, continuity of care, and limiting exposure to severe acute respiratory syndrome coronavirus 2 (SARS-CoV-2) or other infectious agents. Monitoring via video visits may also have a positive impact on the quality of life of patients, resulting in time and cost savings. The following paragraphs briefly describe details on the regulatory and practical aspects of the video visit.

**Table 1:** Expected benefits of video visit implementation for the various stakeholders

Benefit	Patient	Clinician	Hospital
Simplified logistics (reduced traveling)	✓		
Minimized risk of infection	✓	✓	✓
Quality of life	✓		
Potential reduction of the Clinician’s burden and time optimization		✓	✓
Continuity of patient monitoring	✓	✓	✓
Efficient management of visit’s processes (delegation, automation)		✓	
Use of innovative solutions for the optimal management and optimization of patient care			✓
Increased activity volumes (with the same resources)			✓
Formalization of informal clinician-patient interactions through GDPR compliant channels		✓	
Direct and indirect cost reductions (social costs)	✓		✓

### Regulatory aspects and current indications

National guidelines for the use of telemedicine were initially issued in 2014 by the Italian Ministry of Health. On October 27, 2020, in response to the COVID-19 emergency, updated national indications were published and telemedicine is now a fully recognized service offered by the Italian healthcare system [11]. Telemedicine services are currently available in most Italian regions and the COVID-19 crisis has prompted many centers to develop software programs not only for carrying out televisits, but also for uploading documents to be shared between physicians and patients. Indeed, all the documents, including the report of the virtual visit and any prescriptions, have to be shared as an attachment through the platform being used (in compliance with GDPR standards, regional rules and National Law). Printing the prescription could be an option, although in several Italian regions the use of digital prescriptions is already routine, even in a “face-to-face visit” at the hospital. In any case, a printed document could be mailed or delivered whenever required.

According to the new indications, the televisit is defined as a medical service during which the physician and the patient interact remotely in real time via video call, with the patient being visible to the physician during the entire call [11]. This service can be used to provide medical care to patients with a known disease if an in-person physical examination is not required. Prior to attending a video visit, patients must provide written informed consent, which is a straightforward procedure that confirms patient agreement to the televisit. According to Italian indications, from a regulatory and administrative point of view, the televisit is equivalent to the conventional face-to-face visit, with no differences in fees, reimbursement policies, data recording, patient information, and physician responsibility [11]. Notably, physicians are responsible for evaluating whether the televisit was able to achieve the objectives; if these were not met, the physician must prescribe a face-to-face visit. A note concerning the quality of the Internet connection and its appropriateness for performing the televisit must be included in the visit report, along with the usually reported information.

### Video visit model for the follow-up of kidney transplant recipients

#### Profile of eligible patients

The characteristics of kidney transplant recipients who can be followed-up via video visits are summarized in Table 2. Eligible patients should have basic skills in the use of electronic and mobile devices, and be familiar with video call applications. In the absence of such skills, a caregiver or family member can assist the patient during the video call. The clinical condition of eligible patients should be stable in terms of both graft function and immunosuppressive regimen. Uncomplicated patients with a low risk of comorbidities, as well as patients who have been followed-up for at least 12 months, are generally considered good candidates for televisit monitoring. The characteristics listed in Table 2 should not be regarded as strict criteria, and some of them (patient age, time from transplantation) may be extended according to local clinic practices and expertise in virtual monitoring.

**Table 2:** Profile of kidney transplant recipients who can be monitored via video visits

Patient characteristics and clinical status	• 18–70 years old • Stable kidney function • Established immunosuppressive therapy • Low risk of comorbidities • In the follow-up program for ≥ 12 months
Technical requirements	• Access to computer and/or mobile devices • Webcam • Audio and microphone • Absence of firewalls impeding the download and access to teleconference platforms • Good and stable Internet connection • Basic skills in the use of computer/mobile devices and apps for video calling • Presence of a caregiver for assistance if the above skills cannot be ensured

The frequency of televisits should be personalized according to the clinical and personal situation of the patient, health emergency, the distance and ability to reach the center, and the physician’s judgment. We hypothesize at least one face-to-face visit every two televisits.

#### Assessments that can be performed remotely

With the exception of physical examinations, complex instrumental examinations and kidney biopsies that can only be performed at the clinic, all other assessments carried out during a conventional in-person follow-up visit are feasible during a video visit (Table 3). In detail, the following medical actions can be performed remotely: collection of medical history; discussion of clinical parameters including blood pressure, heart rate, and other comorbidity parameters; evaluation of current therapy; prescription of drugs, laboratory and instrumental tests; prescription of specialist evaluation; answering the patient’s questions; writing the video visit report; scheduling upcoming visits.

**Table 3:** Activities of conventional in-person visits that can be performed during the video visit

	Traditional visit	Video visit
Medical history	✓	✓
Assessment of clinical parameters:		
Weight	✓	✓
Blood pressure (BP)	✓	✓
Heart rate (HR)	✓	✓
Drug intake compliance	✓	✓
Other comorbidity parameters (e.g., glucose levels through glucometer for diabetic patients)	✓	✓
Physical examination	✓	×
Evaluation of hematochemical exams	✓	✓
Drug dosage evaluation	✓	✓
Evaluation of instrumental examinations organ’s functionality assessment:		
Kidney US	✓	×
Ecocolordoppler (to be carried out at the TC)	✓	×
MRI	✓	×
Kidney biopsy (to be carried out at the TC)	✓	×
Evaluations of comorbidities:		
Instrumental examinations:	✓	✓
CT scan, MRI, X-ray, US	✓	✓
DXA	✓	✓
ECG	✓	✓
Infectious surveillance	✓	✓
Oncological surveillance	✓	✓
Evaluation/review of the therapy	✓	✓
Drug prescription, hematochemical and/or instrumental exams	✓	✓
Prescription of specialist examinations	✓	✓
Training on pathology, therapy and lifestyle	✓	✓
Scheduling future appointments	✓	✓
Archiving the examination report	✓	✓

#### Video visit organization and medical professionals involved

**Fig. 1:**
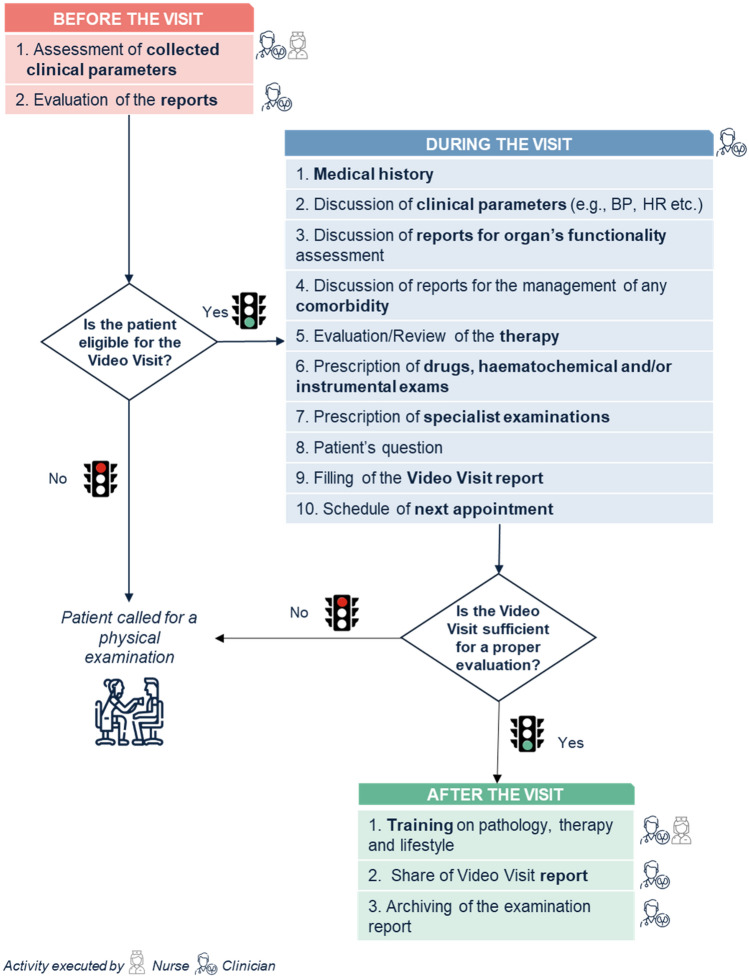
Workflow of the video visit for the follow-up of kidney transplant recipients

Remote monitoring of kidney transplant recipients can be optimized by structuring the process into three steps as depicted in Fig. 1. As a first step, a review of available clinical parameters and previous medical reports will help decide whether a patient is eligible for the video visit or should undergo a conventional in-person visit. Eligible patients will be prescribed a video visit (second step) at the end of which the physician will decide whether remote follow-up was sufficient for proper patient evaluation or if a conventional visit including a physical examination is required. During the video visit the activities listed in Table 3 will be performed as needed. After the video visit (third step), a visit report will be shared with the patient and recorded. Educational material about the condition, therapy, and healthy lifestyle changes may be provided to patients during this phase. Physicians are involved in all steps, while nurses can take over some of the services provided including initial assessment and recording of clinical parameters, and patient education.

#### Supporting tools

To optimize the video visit, patients are invited to collect clinical parameters that can be easily measured at home including body weight, blood pressure, heart rate, and other parameters. To this end and prior to the video visit, a diary will be provided to patients with instructions on how to use it and what information to fill in. Other supporting tools include educational materials to be provided to patients using e-mail or any other document-sharing software.

### Implementation of the video visit

The selection of adequate technology supporting the video visit should involve the information technology (IT) department and considering infrastructure that is already in place. Issues related to data security and privacy also need to be considered. The set-up for the video visit in the physician’s office should consist of a personal computer integrated with, or connected to, a microphone, audio system, and webcam; a stable Internet connection; and firewall settings that allow access to free software for video-communication. The choice of video-communication software should take the following criteria into consideration: known by the users and widely used in everyday life; free; easy to use; patient registration not needed to participate in the video call; no need for the patients to download any additional software; and document sharing should be supported. The televisit model has been designed by the Expert Panel with the aim of avoiding technical limitations, allowing its implementation regardless of the digital platform, and extending its adoption to as many centers as possible. Should no proprietary platform be in place in the Center, the group suggests the most suitable platform (according to GDPR and other requirements) among those freely available on the market.

With regard to security and privacy issues, personal information collected during the video visit is considered equivalent to that collected during a conventional in-person visit and will be treated in accordance with current regulations on the protection of sensitive data. The data protection office and the IT department of the clinic should make sure that the selected video-calling platform manages personal data according to current regulations.

## The video visit model: discussion and conclusions

The proposed model of video visits for the follow-up of kidney transplant recipients was based on the general structure of a conventional in-person visit and translates most of the medical actions provided by clinicians during such check-up visits into virtual mode. Overall, the suggested real-time video visit appears to be a feasible, adaptable, and easy-to-implement option in the setting of transplant recovery in Italy. This option may alleviate the increasing organizational burden on transplant and nephrology clinics and, at the same time, ensure uninterrupted and timely patient follow-up despite the restrictions imposed by the COVID-19 pandemic and other healthcare crises. The video visit, however, does not replace conventional in-person visits, which continue to be indicated in many cases. Rather, it should be regarded as complementary to current follow-up programs. Importantly, according to current national indications for telemedicine [11], physicians are expected to evaluate the quality and results of the video visit and to prescribe a face-to-face visit in case of insufficient patient evaluation and assistance.

The healthcare crisis caused by the COVID-19 pandemic has no doubt contributed to the increased interest in telemedicine. We currently do not know what the role of telemedicine services implemented during the pandemic will be once the crisis is over. As the quality and the services that can be offered via video visit are steadily improving, we expect telemedicine to achieva a stable position in the management of patients with chronic conditions. Concerns have been raised that telemedicine may be precluded to patients who cannot afford the devices and Internet connection required for the video visit. In this respect, we estimate that the savings related to the decreased traveling to the clinic may largely offset the costs of devices and connection.

With regard to the selection of kidney transplant recipients who can be adequately followed-up via video visits, we propose involving patients in the age range 18–70 years, with an overall stable condition, low risk of comorbidities, and who have been in a post-transplant follow-up program for at least 12 months, in agreement with current national indications that discourage the use of telemedicine in cases of acute disease and fragility [11]. The suggested criteria are however adjustable to the telemedicine competency and expertise of both the center and the patients and may change to include more complex patients as our knowledge about the outcomes of remote monitoring of kidney transplant recipients increases. The risks associated with the visit to the healthcare facility must always be taken into account and evaluated for each patient. Groups of patients that are not included within the general criteria may be positively evaluated (i.e., children when the parents provide consent, or older subjects with proven ability to sustain a video call either alone or with the support of a caregiver). Interestingly, a recent small survey among health care providers revealed that stable health status and a well-established patient-nephrologist relationship are regarded as key factors for the success of remotemonitoring of kidney transplant recipients [12]. According to the interviewed health care providers, it is advisable to initiate telemedicine-based monitoring on a small scale and with selected patients in order to achieve good results [12].

Studies assessing the feasibility and effectiveness of remote monitoring of kidney transplant recipients are still limited. We currently do not know whether and to what extent the benefits of video visits anticipated in Table 1 will be confirmed in clinical practice. The first randomized clinical trial evaluating conventional versus telemedicine-based follow-up in 46 kidney transplant recipients during the first year after transplantation found significantly better compliance in patients monitored remotely compared with those attending in-person visits [13]. The study also highlighted a positive effect of telemedicine on medical service utilization and costs [13, 14]. Evidence from small, qualitative studies assessing the impact of telemedicine in the follow-up of kidney transplant recipients is also available [2, 12, 15, 16]. Moreover, we cannot exclude that implementation of the video visit will also result in a reduction in transport-associated carbon emissions [17]. The overall picture emerging from these reports is that telemedicine is well-accepted by patients, with the possibility of alternating video and in-person visits being particularly appreciated [12]. Perceived benefits in terms of time and cost savings have also been reported [2, 12, 15]. The available reports on remotemonitoring of kidney transplant recipients during the COVID-19 pandemic have consistently shown that telemedicine-based follow-up, regardless of the technological set-up that was used, ensured prompt interventions, continuity of care, and safe management of patients [3, 4].

As with other approaches of telemedicine and self-care at home, our video visit model requires patients to be actively involved in their follow-up, as they are invited to provide clinical parameters that are easily measurable at home and information about compliance to prescribed medication in a diary. Blood pressure can now be easily measured in non-hospital settings.. Furthermore, the possibility of booking an appointment ensures safety conditions even during the COVID-19 pandemic.

BP monitoring at home using Ministry of Health approved medical devices is also an option. At the transplant center, both the patient and a caregiver (i.e., a family member) are actually trained to use the available validated tools, and are provided with all the information on diet and drug dosage/administration. This may contribute to patient empowerment with a potential benefit in terms of compliance to prescribed therapies and recommended lifestyle changes, improvement of visit quality and patient-physician communication. In line with this expectation, a recent survey among kidney transplant recipients and healthcare professionals conducted in Denmark to evaluate an app and workflow for follow-up found increased collaboration and preparation of patients during the visits and improved dialogue between patients and clinicians [16].

Obviously, the main drawback is the impossibility to perform an “in person” classical examination. The televisit model necessarily implies that the patient is stable. Televisits should be considered as complementary to, and not a replacement for, a face-to-face visit. It is also the physician’s responsibility to recall an outpatient visit any time one is needed on the basis of the patient’s clinical status (Legal requirements).

All patients must be assessed for eligibility in order to undergo a televisit, and fragile patients (psychologically or mentally vulnerable individuals) are carefully considered for participation, evaluating the availability of a feasible caregiver. The televisit should be an opportunity to improve the follow-up of a kidney transplant recipient without replacing the onsite visit. Whenever the patient or the physician needs a face-to-face examination, this is discussed and arranged.

Moreover, we acknowledge that explicit patient input is missing. However, we underline that in “real life” experience, besides onsite visits, an increasing proportion of patients have additional contacts with the physicians and nurses of the transplant team through phone calls, e-mails and phone text/voice messages. In this view, the televisit represents an organized and privacy-law respectful tool to fulfill the patient’s unmet needs, thus responding to an implicit but obvious input. Indeed, the patient’s point of view and needs were always central during the discussion among the panelists, as all of them have extensive experience and are constantly in touch with the patients, and are thus aware of the main needs and the most important practical issues. The aim of the panel was to first define a consensus model to be further shared with the patients. A second phase of this project is expected and will involve extensive testing of the model in the field, with the opportunity to collect feedback and suggestions on areas of possible improvement both from patients/caregivers and physicians.

In conclusion, the video visit model for the remote monitoring of kidney transplant recipients suggested herein is simple and adaptable and should be easy to adopt by transplant and nephrology clinics with some telemedicine infrastructure, as well as by most transplant patients. The video visit is not intended to replace face-to-face visits, but is meant as an additional tool for improving the follow-up of kidney transplant recipients, and to be integrated into current monitoring protocols.

## Data Availability

Not applicable.
